# An unprecedented view of ocean currents from geostationary satellites

**DOI:** 10.1038/s41561-026-01943-0

**Published:** 2026-04-13

**Authors:** Luc Lenain, Kaushik Srinivasan, Roy Barkan, Nick Pizzo

**Affiliations:** 1 Scripps Institution of Oceanography, UCSD, La Jolla, CA USA; 2 Atmospheric and Oceanic Sciences, UCLA, Los Angeles, CA USA; 3 Department of Geophysics, TAU, Tel Aviv, Israel; 4 Graduate School of Oceanography, URI, Narragansett, RI USA

**Keywords:** Physical oceanography, Physical oceanography

## Abstract

Oceanic submesoscale currents dominate the vertical exchanges of heat, biological nutrients and carbon between the shallow and the deep ocean and strongly influence the lateral dispersion of biogeochemical tracers and pollutants. Observing these surface intensified currents, however, has been a long-standing challenge due to their small scales and rapid evolution. Here we introduce Geostationary Ocean Flow (GOFLOW), a deep learning framework that takes advantage of geostationary satellites’ contiguous sequences of thermal imagery to produce hourly, high-resolution surface velocity fields that capture submesoscale circulations. Our approach does not assume simplified dynamical balances and inherently filters internal wave noise, both of which limit state-of-the-art satellite altimetry. Applying GOFLOW to the Gulf Stream, we provide satellite-based measurements of submesoscale current statistics, revealing characteristic asymmetries in vorticity and divergence previously documented only in high-resolution circulation models. This ability to routinely map the ocean’s energetic submesoscale currents provides a transformative data source to advance Earth system forecasting, to mitigate ocean pollution, to monitor marine ecosystems and to reduce climate model uncertainties.

## Abstract (web summary)

A deep learning framework called GOFLOW enables observation, and may ultimately improve forecasting of, oceanic surface currents down to submesoscale spatial and temporal resolutions from thermal imagery routinely collected by geostationary satellites.

## Main

Due to the sparsity of in situ measurements, coincident, high-resolution, global observations of the ocean surface velocity field represent a long-standing challenge in physical oceanography^1,2^. These surface currents control the flux of mass, momentum and heat, interact with the atmospheric circulation and strongly modulate Earth’s weather and climate and the transport of marine debris and biogeochemical tracers^3^. Hindering progress is the large range of scales needed to understand and interpret oceanic flows—from oceanic basin scales that contain features that last months, to kilometre-scale currents that evolve over the course of a day.

The TOPEX/Poseidon altimetry mission^4^, often considered one of the most successful ocean experiments of all time^5^, provided the first global mapping of ocean surface topography that revolutionized our understanding of ocean circulation and, in turn, its impact on global climate. This has led to consistent, global measurements of oceanic sea surface height (SSH) at spatial scales on the order of hundreds of kilometres and larger, and with a repeat cycle of approximately 10 days, through the launch of multiple international satellite altimetry missions. Geostrophic balance^6^ can then be used to determine the associated surface velocity field at comparable spatio-temporal scales. This velocity field is regularly being used as an assimilation product for weather and climate models^7^.

In the last decade, it has become evident that smaller scale currents that cannot be resolved by traditional altimetry capabilities dominate the vertical exchange of properties, such as heat, CO_2_ and nutrients, between the ocean surface and its interior^8–11^, and strongly control the lateral dispersion of buoyant materials and pollutants^12,13^. These surface-intensified submesoscale currents (SMCs) consist of anisotropic fronts, filaments and eddies that rapidly evolve on timescales on the order of a day and with spatial scales between hundreds of metres and tens of kilometres, and exhibit substantial ageostrophic flow characteristics^14–16^. As a result, measuring their dynamic and kinematic properties requires specially designed field campaigns^17–19^, but those only allow for point-like measurements and snapshots in time and cannot offer global coverage. The recently launched Surface Water and Ocean Topography (SWOT) mission now provides global submesoscale SSH variance at unprecedentedly high spatial resolution^20^. However, its reliance on SSH snapshots presents two fundamental challenges: first, the 21-day repeat cycle cannot resolve the rapid evolution of submesoscale currents; and second, the SSH signal intrinsically contains energetic internal tides and waves. Separating this wave-induced variability from the balanced and frontal circulation is a complex dynamical problem that is the subject of ongoing research, complicating the inference of surface currents.

Recent advances in machine learning-based reconstruction have attempted to overcome some of these limitations. SSH reconstruction has been enhanced by fusing altimetry with sea surface temperature data^21–28^, in turn improving the resolution of mesoscale features down to 50–70 km scales. Others have used neural networks to extract finer-scale currents from SSH measurements while filtering out internal wave contamination^29,30^. However, all these methods remain fundamentally constrained by two factors: the sparse temporal sampling of orbital platforms (10–21-day revisit cycles) and their reliance on geostrophic assumptions. They can improve spatial resolution and filter noise but cannot capture the rapid evolution of submesoscale features that change on daily timescales unless these same techniques are applied to geostationary satellites.

Here we describe Geostationary Ocean Flow (GOFLOW), a novel approach to observe oceanic surface currents at submesoscale spatial and temporal resolutions of kilometres and hours by employing readily available global sea surface temperature data from geostationary satellites together with U-Net machine learning techniques trained on state-of-the-art global ocean models. We demonstrate GOFLOW’s ability to provide detailed maps of surface velocity and velocity-gradient information from basin scales to submesoscales at unprecedented resolution. We further compute the first satellite-based measurements of submesoscale current statistics that confirm previous findings from high-resolution oceanic circulation models, thereby exemplifying the vast scientific and operational potential of GOFLOW.

## Surface currents derived from geostationary satellites

Historically, efforts to derive surface velocity from sequential satellite imagery of a scalar such as sea surface temperature (SST) have fallen into three main categories: optical flow^31^, feature-tracking methods^32–34^ and dynamical methods^35–39^. Each of these approaches faces substantial practical and physical limitations^40^.

Optical flow algorithms attempt to estimate motion by directly inverting the conservation equation for the advected scalar—in this case, SST^41^. This is a notoriously challenging inverse problem. Its accuracy is compromised by non-advective sources and sinks, chief among them the strong diurnal heating and cooling from solar radiation and air–sea heat fluxes, which can muddle the signature of horizontal advection. Alternatively, feature-tracking methods circumvent the need to invert the full advection equation by instead identifying and tracking distinct thermal features, or fronts, between successive images^32^. Fronts, characterized by strong horizontal temperature gradients, are reliable tracers of the underlying flow. The primary limitation of this approach, however, is the relative sparsity of such strong features. Whereas major currents such as the Gulf Stream are associated with persistent, large-scale fronts, the broader ocean surface often lacks well-defined features, resulting in sparse and incomplete velocity estimates.

Dynamics-based methods have also been employed to extract velocities directly from SST, most notably through inversion of the surface quasigeostrophic potential vorticity equation^35,37,38^. While effective at large scales (typically >50 km), this approach faces two fundamental limitations at submesoscales. First, the quasigeostrophic approximation itself breaks down as ageostrophic motions become important. Second, the Poisson-based inversion requires spatially complete data without gaps, a condition readily met by low-resolution, cloud-penetrating microwave SST observations, but severely limiting for high-resolution infrared imagery such as Geostationary Operational Environmental Satellites (GOES), where clouds create frequent spatial discontinuities.

**Fig. 1:**
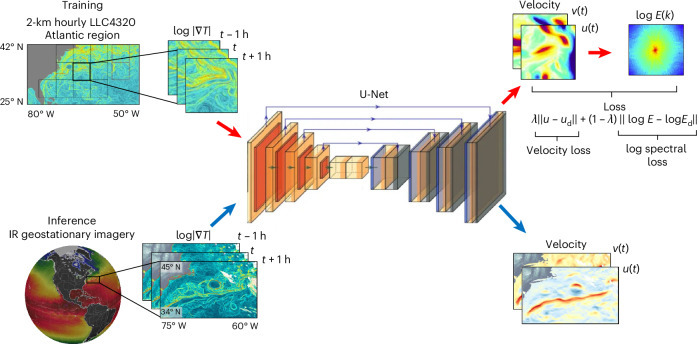
Flow chart of the GOFLOW product. GOFLOW is trained on 2-km hourly global Latitude–Longitude–polar Cap 4320 (LLC4320) 1/48^o^ MIT General Circulation Model (MITgcm) solution in the Atlantic Ocean over 5.3^o^ × 5.3^o^ boxes. Three snapshots at times *t* − 1 h, *t* and *t* + 1 h are used to generate velocity fields (*u*, *v*) at time *t* with an associated log of their energy wavenumber spectra (with *k* representing wavenumber and *E* the kinetic energic spectrum). These are used to establish a loss function $\lambda | | u-{u}_{{\rm{d}}}| | +(1-\lambda )| | \log E-\log {E}_{{\rm{d}}}| |$ where the subscript d denotes data and *λ* is a weighting parameter. The inference data is a series of orbital infrared geostationary imagery, input as the logarithm of the gradient of the temperature, with an output of a velocity field. Land topography is from ETOPO 2022^63^.

**Fig. 2:**
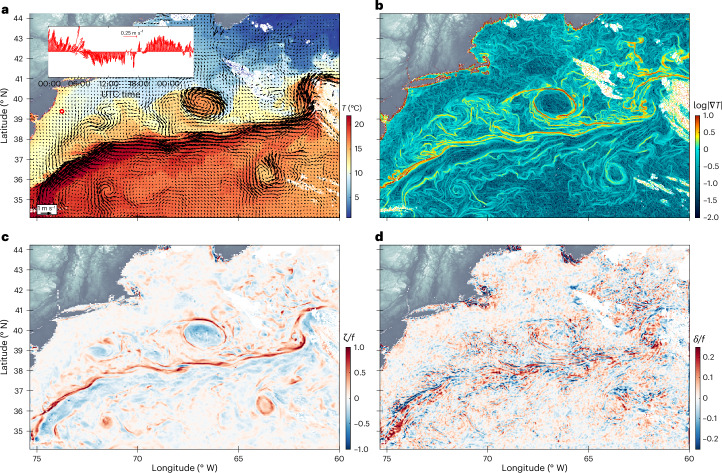
GOFLOW velocity product and its gradients computed in the Gulf Stream region in the Atlantic Ocean. **a**, GOFLOW velocity field, overlaid on top of SST measured by GOES-East^64^ on 13 April 2023 at 18:30 UTC. The inset shows a time series of the velocity field over the course of 30 hours at the location indicated by the red diamond, revealing a pronounced modulation of the surface currents by inertial oscillations induced by Earth’s rotation. Land topography is from ETOPO 2022^63^. **b**,**c**, The logarithm of the temperature gradient magnitude, $\log | \nabla T|$ measured by GOES (**b**) and the GOFLOW vertical vorticity component *ζ* (normalized by the Coriolis parameter *f*), remarkably delineating the Gulf Stream and associated eddy field (**c**). **d**, The GOFLOW normalized horizontal divergence *δ*/*f*, a field that cannot be measured or inferred from traditional altimeters. Note the close correlation between the Gulf Stream velocities derived from GOFLOW and the GOES SST field.

The GOFLOW algorithm (Fig. 1) introduces a paradigm that builds on the strengths of feature tracking while overcoming its fundamental limitations through two key innovations. First, instead of relying solely on strong, easily identifiable fronts, we enhance the visibility of the entire frontal network by using the logarithm of the temperature gradient magnitude, $\log | \nabla T|$, as our input field. This transformation amplifies the signature of the numerous weak fronts that are densely distributed throughout the flow domain, turning a sparse set of tracers into a rich, full-field source of kinematic information (for example, Fig. 2b). Second, we leverage deep learning to interpret the complex evolution of this dense frontal network. Extracting a velocity field from the subtle, non-local motion of countless intertwining fronts is a task ill-suited to traditional correlation-based tracking algorithms.

**Fig. 3:**
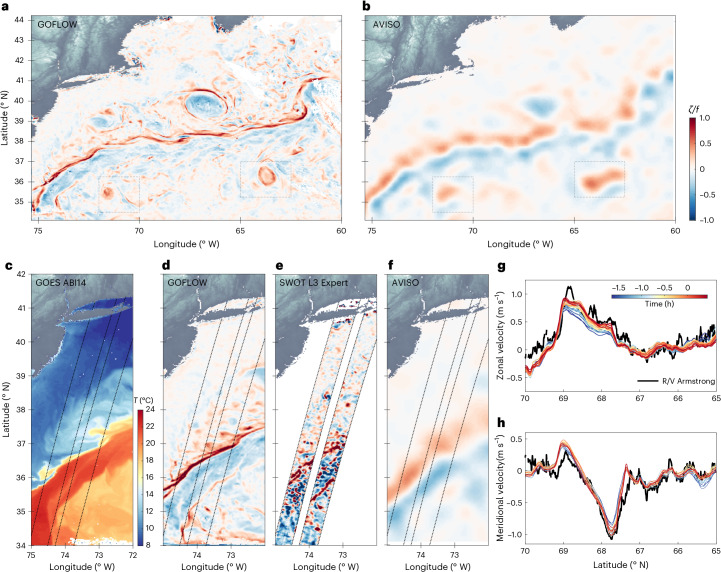
Comparison of GOFLOW velocity and vorticity field with other state-of-the-art surface current measurements and estimates in the same region as in Fig. 1. **a**, The normalized vertical component of vorticity *ζ*/*f* computed from the GOFLOW product. **b**, The geostrophic *ζ*/*f* field derived from SSH measurements from the AVISO SSALTO/DUACS programme^65^. **c**, Temperature from GOES^64^: note the sharp temperature contrast on the north wall of the Gulf Stream and the associated meanders. **d**–**f**, *ζ*/*f* computed from GOFLOW (**d**) the recently launched SWOT satellite (L3 Expert product) (**e**) and AVISO SSALTO/DUACS (**f**). Dashed lines across **d**–**f** mark SWOT overpass tracks, for reference. **g**,**h**, Comparison of zonal (**g**) and meridional velocity (**h**) components between a couple of hours of GOFLOW estimates (referenced to 15 May 2023 12:02 UTC) and in situ observations (solid black line; at 20–25-m depth range) collected from the R/V Armstrong on 15 May 2023^66^ (Extended Data Fig. 1 and Methods). Note that the SWOT L3 products used here are preliminary and may not reflect the final accuracy of SWOT.

We therefore employ a U-Net^42^, a standard fully convolutional neural-network architecture trained to read in a sequence of three consecutive hourly $\log | \nabla T|$ snapshots and output the instantaneous surface velocity field at the central time (Fig. 1). By learning from sequential data, the network implicitly learns the dynamics of advection; it learns how frontal patterns deform and displace over time in response to an underlying velocity field. Crucially, the U-Net is trained exclusively on data from a high-resolution ocean general circulation model (MITgcm LLC4320; ref. ^43^) within geographically restricted 5.3° × 5.3° sub-domains. This strategy reduces the likelihood of the network learning large-scale circulation biases of the ocean model (for example, the incorrect mean path of the Gulf Stream^44^) and instead helps it focus on the generalized physics of advection of temperature gradients by the turbulent velocity field. The result is a model that can be applied to real geostationary satellite data, inferring surface velocity with an unprecedented degree of accuracy and spatial resolution (Figs. 2 and 3).

From a statistical perspective, the U-Net can be viewed as learning an approximation to the conditional probability distribution *p*(**u** ∣ *T*_*t*−Δ*t*_, *T*_*t*_, *T*_*t*+Δ*t*_). In the training data (LLC4320), this relationship is probably shaped by the physics of advection and is expected to be more transferable than learning the unconditional distribution *p*(**u**), which could reflect the specific climatological biases of the underlying ocean model. Furthermore, U-Net is a fully convolutional neural network, making it spatially agnostic (more specifically, translationally equivariant) and allowing for seamless application from small training patches to entire satellite domains during inference. By implicitly tracking fronts of all scales and strengths, GOFLOW reveals fine-scale ocean currents at unprecedented detail (spatial scales of the order of approximately 10 km; Fig. 2c,d), and with remarkable temporal resolution (red diamond and associated time series in Fig. 2a).

## GOFLOW product validation

A compelling demonstration of GOFLOW’s capabilities is evident when its vorticity field is compared with that computed from the commonly used geostrophic current product of Archiving, Validation and Interpretation of Satellite Oceanographic Data (AVISO) (Fig. 3a,b). The contrast is striking: GOFLOW captures sharp eddy and frontal structures, whereas the AVISO-derived vorticity field appears diffused and smoothed, a result of its 10-day averaging window that filters out short-lived oceanic features. Two regions, delineated by boxes, highlight these differences. In the GOFLOW output, small eddies are clearly defined and spatially coherent; in contrast, these structures are difficult to discern in the AVISO field, with both their intensity and position differing substantially.

The GOES SST field reveals the intricate meandering of the Gulf Stream, including filaments and embedded eddies that align closely with features identified in the GOFLOW vorticity field (Fig. 3c,d). This spatial correlation is the hallmark of submesoscale turbulence, which has mainly been displayed in numerical simulations^45–47^.

This specific region is selected because it coincides with a SWOT satellite overpass, allowing us to further compare the high-resolution SWOT geostrophic vorticity with GOFLOW and AVISO (Fig. 3d–f). It appears that the SWOT vorticity estimates are noisier and less coherent compared with GOFLOW’s consistent and well-organized structures. This discrepancy is probably attributable to internal wave contamination in the raw SWOT SSH signal, which is not easily filtered and can be aliased into the final velocity product. GOFLOW circumvents this issue, as the horizontal advection of temperature by internal waves is weak^48^, meaning our methodology inherently and physically filters out this source of noise.

**Extended Data Fig. 1:**
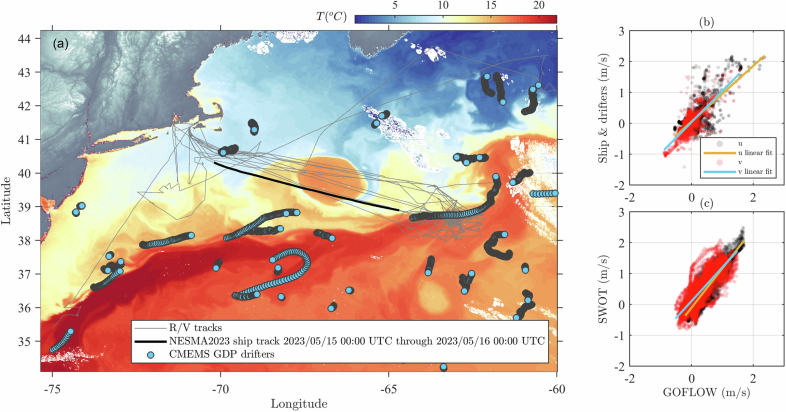
Study region and collocated comparisons between in situ (drifter/ship) and SWOT-derived surface currents. (**a**) Locations of GDP drifters and research vessels (R/V) tracks considered in the comparison in panel (**b**), including the one used in Fig. 3, overlaid on a sea surface temperature (SST) image acquired by GOES-East on April 13, 2023, for reference. (**b**) In-situ collocated drifters and ship-based zonal u and meridional *v* surface current observations, and (**c**) SWOT L3 Expert products. Note, the GOFLOW products presented in (**c**) are computed using data collected coincident with the SWOT overpass that occurred on April 13 2023 at approximately 02:30 UTC. We find rmse of 0.27 and 0.24 m/s for the in situ and SWOT observations, respectively, and coefficient of determination r^2^ for (u,v) of (0.69,0.73) for panel (**b**) and (0.86,0.81) for panel (**c**). Results presented in (**b**) use representative subsets of observations across all seasons with their geographical extent also plotted in (**a**).

To directly validate GOFLOW, we compare its predicted zonal and meridional surface velocities with independent in situ ship-based Acoustic Doppler Current Profiler (ADCP) measurements (Fig. 3g,h), collected during the Office of Naval Research-funded New England Seamounts Experiment (NESMA) 2023 campaign (Extended Data Fig. 1a and Methods). The close agreement between GOFLOW and these independent observations highlights its ability to generate a high-resolution velocity field that matches, and in some respects exceeds, existing satellite products. An additional comparison of GOFLOW with drifters and shipboard measurements spanning all seasons, along with a comparison with SWOT observations collected during the CalVal orbit are provided in Extended Data Fig. 1b,c.

Furthermore, a model-specific check comes from known circulation biases in the training simulation. In the Gulf Stream separation region, the LLC4320 solution has been shown to separate the current too far south relative to AVISO-based estimates of the mean dynamic topography and associated jet path (for example, refs. ^44,49^). In our Gulf Stream domain, by contrast, the GOFLOW vorticity ridge and velocity maxima are closely locked to the sharp temperature front visible in the GOES imagery (Figs. 2 and 3), and they follow the observed Gulf Stream path inferred from SST and AVISO rather than exhibiting a clear southward displacement. Taken together with the ADCP and drifter comparisons, this supports our working hypothesis: training on geographically restricted sub-domains and on the conditional map *p*(**u** ∣ *T*_t−Δt_, *T*_t_, *T*_t+Δt_) helps limit the imprint of large-scale model climatological errors and encourages the network to focus on the local advection of temperature gradients.

## Observations of submesoscale turbulence statistics in the Gulf Stream

**Fig. 4:**
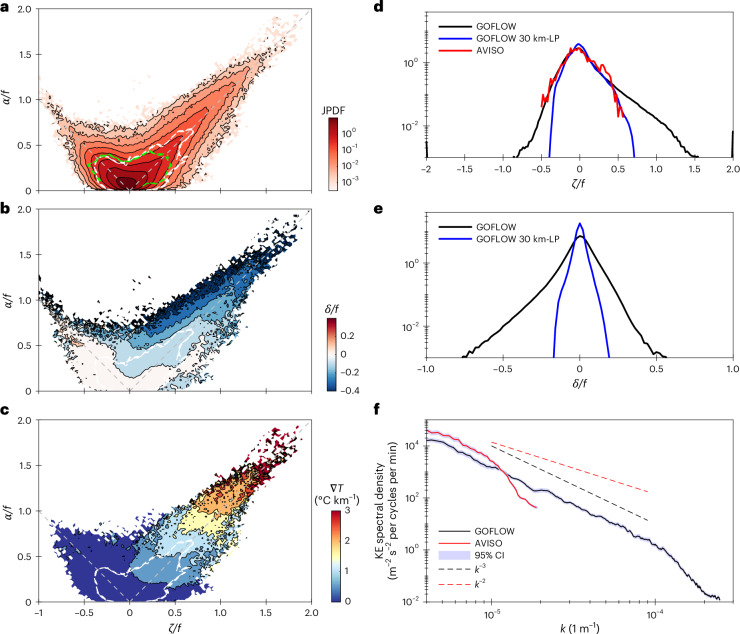
Flow kinematics and statistics computed over the Gulf Stream region from GOFLOW and AVISO. **a**, The joint probability density function (JPDF) of normalized strain (*α*/*f*) vs normalized vorticity *ζ*/*f*. **b**,**c**, The conditional average of horizontal divergence (*δ*/*f*) (**b**) and horizontal temperature gradient magnitude (∣ ∇ *T*∣) (**c**) on the strain–vorticity distribution. White dashed contour lines in **a**–**c** show the contribution to these JPDFs from spatial scales greater than 30 km (using anisotropic uniform low-pass filter), and the green dashed contour lines in **a** show the corresponding AVISO JPDF, highlighting that large positive vorticity, strong negative horizontal divergence and large temperature gradients are dominated by smaller scale (submesoscale) motions. **d**,**e**, The 1D PDF of *ζ*/*f* (**d**) and *δ*/*f* (**e**) computed from GOFLOW (black), GOFLOW low-passed filtered at 30 km (blue) and AVISO (red), confirm consistency in the large-scale dynamics and emphasize that only GOFLOW captures the most intense vorticity and horizontal divergence events (the tails of the distributions). **f**, Kinetic energy power spectral densities computed from GOFLOW (black) and AVISO (red) demonstrate the extensive range of smaller scales resolved by GOFLOW, with dashed red and black lines showing *k*^−2^ and *k*^−3^ spectral slopes, respectively. GOFLOW statistics are computed in a 12.8^o^ × 6.6^o^ region on 13 April 2023 from 16:30 UTC to 20:30 UTC. AVISO statistics are computed over the same region using the corresponding daily SSALTO/DUACS product.

The key dynamical properties of oceanic SMCs that distinguish them from larger scale, geostrophically balanced motions are associated with their prominent ageostrophic circulation patterns. These ageostrophic flows, which are often characterized by divergent and convergent motions, drive the formation of temperature fronts, alter ocean energetics and modify the vertical and horizontal transport of biogeochemical tracers and pollutants^9,12,13,47,50–52^. A common way to identify the significance of such ageostrophic effects is through the asymmetry and skewness they lead to in 1D and joint probability density functions (PDFs) of various flow properties^16,50,53–56^. The high-resolution GOFLOW velocity field provides a unique opportunity to produce such statistics throughout the Gulf Stream region from satellite data (Fig. 4).

The joint PDF of normalized strain (*α*/*f*) and vorticity (*ζ*/*f*) and the 1D vorticity and horizontal divergence PDFs indeed show a pronounced skewness towards high strain, high positive vorticity and high negative divergence values that are demonstrably associated with motions of scales smaller than 30 km (Fig. 4a, dashed white line; Fig. 4d,e, black and blue lines). Such asymmetries cannot be captured by the coarser, geostrophic-based AVISO estimates (Fig. 4a, dashed green line; Fig. 4d, red line).

These ageostrophic SMC signals exhibit the strongest temperature gradients (Fig. 4c) and convergence values (particularly where *α*/*f* > *ζ*/*f* > 0; Fig. 4b), confirming in observations dynamical relationships that have only been illustrated in numerical solutions^55^ (Extended Data Fig. 8).

Furthermore, because the GOFLOW velocity product does not depend on the geostrophic assumption, it provides the first satellite-based statistical representation of the oceanic horizontal divergence field (Fig. 4b,e), which is responsible for driving vertical exchange processes^8,15^ and for modulating air–sea interactions^57^.

**Extended Data Fig. 2:**
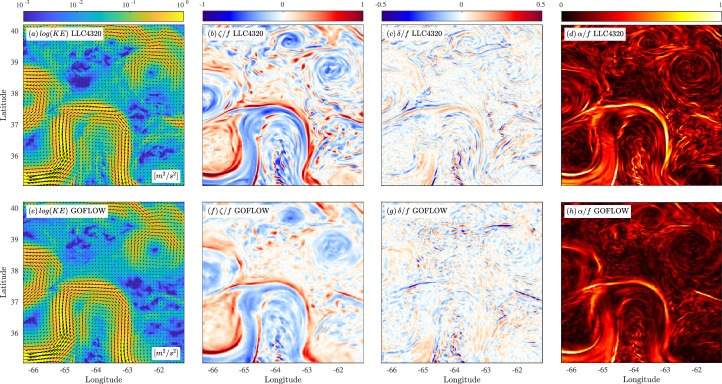
A comparison on the held-out validation set between ground-truth LLC4320 (top panels) and the GOFLOW reconstruction (bottom panels) for a representative snapshot. (**a,e**) Logarithm of KE with arrows showing velocity vectors, (**b,f**) *ζ*/*f*, (**c,g**) *δ*/*f*, and (**d,h**) *α*/*f*.

**Extended Data Fig. 3:**
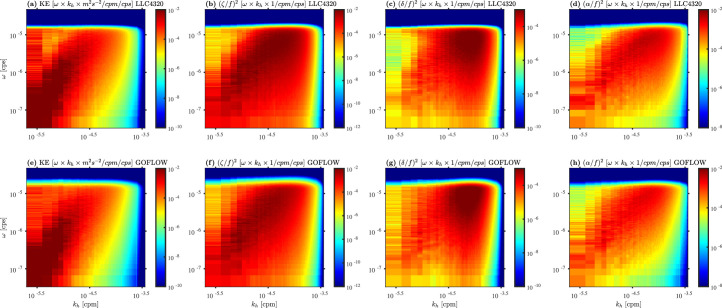
Frequency-wavenumber PSD comparison between the held-out validation set in LLC4320 (top panels) and the corresponding GOFLOW reconstruction (bottom panels). (**a,e**) KE PSD, (**b,f**) *ζ*/*f* variance (normalized enstrophy) PSD, (**c,g**) *δ*/*f* variance PSD, and (**d,h**) *α*/*f* variance PSD. All PSDs are computed using 3D FFT in (*x*, *y*, *t*), transformed to polar coordinates (*r*, *θ*, *t*), and then averaged in the *θ* direction to provide a frequency radial-wave number(*ω*, *k*_*h*_) PSD. A Hanning window is applied in all directions prior to carrying out the FFT. For visual purposes, all PSDs are multiplied by *ω*x*k*_*h*_ before plotting to compensate for the logarithmic stretching.

**Extended Data Fig. 4:**
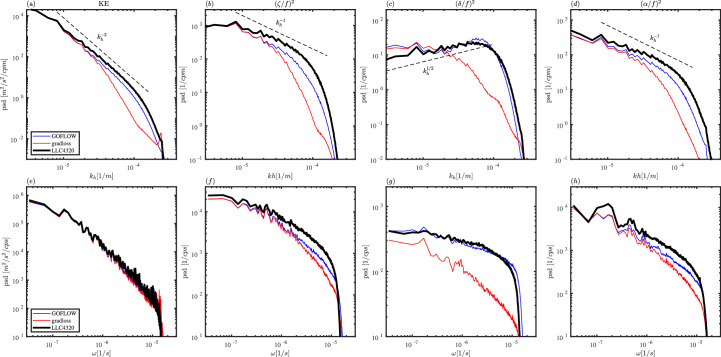
A comparison of (**a-d**) the radial-wavenumber (*k*_*h*_) power spectral densities (PSDs) and (**e-h**) frequency (*ω*) PSDs computed from LLC4320 (solid black lines), gradloss reconstruction (solid red lines; see Methods section 4 for details), and the GOFLOW reconstruction (solid blue lines). (**a,e**) Kinetic energy (KE) PSDs, (**b,f**) *ζ*/*f* variance (normalized enstrophy) PSDs, (**c,g**) *δ*/*f* variance PSDs, and (**d,h**) *α*/*f* variance PSDs. All PSDs are computed using 3D FFT in (*x*, *y*, *t*), transformed to polar coordinates (*r*, *θ*, *t*), and averaged in the *θ* direction to provide a frequency radial-wave number (*ω*, *k*_*h*_) PSD (see Extended Data Fig. 3), and then integrated in *ω*(*k*_*h*_) to produce radial-wavenumber (frequency) PSDs. A Hanning window is applied in all directions prior to carrying out the FFT.

**Extended Data Fig. 5:**
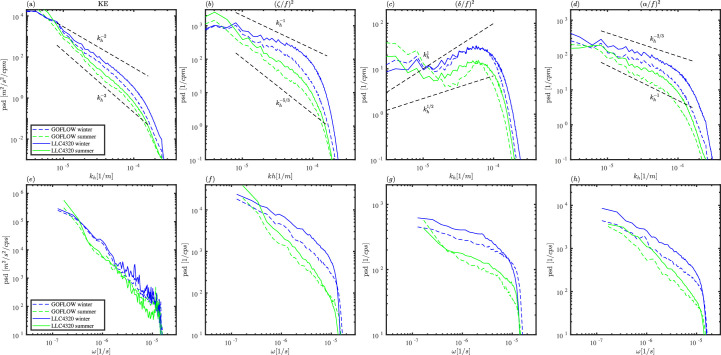
Same as Extended Data Fig. 4 but comparing the seasonal variations between winter (blue lines; January-March) and summer (green lines; July-September). GOFLOW is able to reproduce the seasonal variation present in LLC4320 with respect to spectral slopes and energy levels. It matches better in summer than in winter.

**Extended Data Fig. 6:**
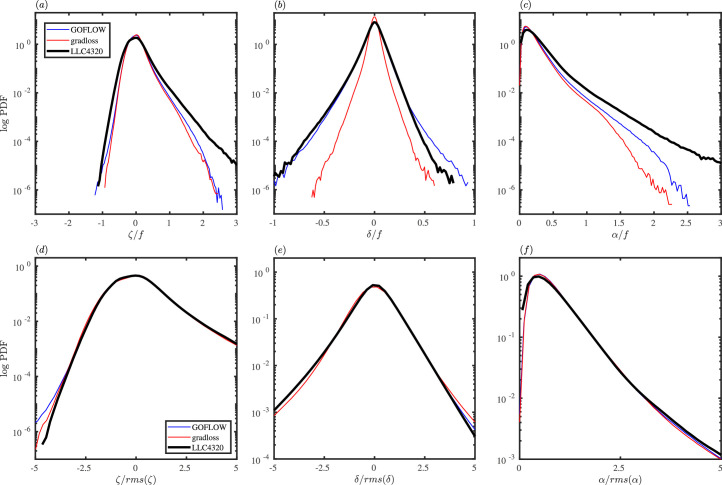
A comparison of the logarithm of the probability density functions (PDFs) between LLC4320 (solid black lines), gradloss reconstruction (solid red lines; see Methods, section 4 for details), and the GOFLOW reconstruction (solid blue lines). Top panels show the PDF of (**a**) normalized vorticity *ζ*/*f*, (**b**) normalized horizontal divergence *δ*/*f*, and (**c**) normalized strain-rate variance *α*/*f*. Bottom panels show PDFs of the same fields as in (**a-c**), but the fields are normalized by their root-mean-square (rms) values. The corresponding PDFs (**d-f**) therefore highlight differences in the shapes of the distributions that are not a result of differences in the rms values.

**Extended Data Fig. 7:**
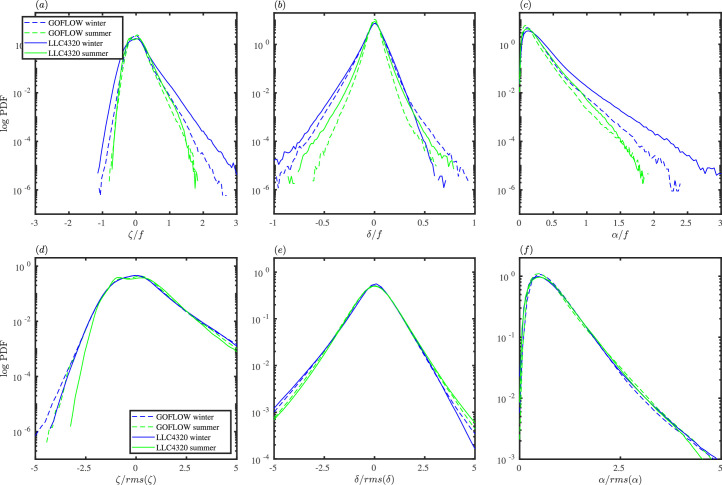
Same as Extended Data Fig. 6, but comparing the seasonal variations between winter (blue lines; January-March) and summer (green lines; July-September). GOFLOW is able to reproduce the seasonal variation present in LLC4320, particularly for the enhanced skewness in the PDFs in winter compared with summer.

The kinetic energy spectrum of GOFLOW (black line; Fig. 4f) spans nearly two decades of wavenumbers and displays a spectral slope of ≈*k*^−3^, consistent with previous ship-based estimates^58^. There is a sharp drop in variance around ~10-km scales. This drop-off scale is consistent with the effective resolution of the LLC4320 solution the U-Net is trained on (that is, the scales unaffected by numerical dissipation; Extended Data Fig. 4a) and probably represents GOFLOW’s effective resolution as well. Numerical simulations with prominent skewness in the PDFs (for example, Extended Data Fig. 6), comparable to that shown here, are typically characterized by a shallower spectral slope, ≈*k*^−2^ (refs. ^45,47,50,59^). This emphasizes that determining the underlying dynamics purely based on measured spectral slopes can be highly misleading. In agreement with previous findings (for example, ref. ^60^), we find appreciable seasonality in LLC4320 and in the GOFLOW reconstruction, with shallower spectral slopes and larger skewness in winter, when SMCs are more energetic (compared to summer; Extended Data Figs. 2, 3, 5 and 7 and Extended Data Table 1).

In summary, these statistical views provide a compelling synthesis: GOFLOW not only resolves submesoscale features in space and time, it also seems to capture their statistical signatures and dynamical characteristics and their potential role in mediating air–sea coupling.

## Discussion

We have presented novel high-resolution, space–time reconstructions of ocean surface currents and their derivatives, obtained from readily available geostationary satellite observations. These reconstructions, made possible through the GOFLOW framework, reveal a level of detail of the velocity field and its kinematics that surpasses the capabilities of existing global products such as AVISO and SWOT.

By capturing not just the velocity field but also its gradients, GOFLOW resolves ocean currents across a broader and finer range of spatial scales than has previously been possible, providing both the basin-scale circulation that sets the general flow structures and material environment (for example, the Gulf Stream) and the detailed spatio-temporal evolution of submesoscale features.

A central feature of GOFLOW is the high temporal resolution of the geostationary satellite infrared imagery. With updates every hour (used here, this could be at even higher frequency), the product provides a dynamic view of the ocean surface that is orders of magnitude more frequent than the AVISO daily mean or the SWOT 21-day revisit cycle. This temporal resolution, together with GOFLOW’s ability to estimate the ageostrophic flow component, enables tracking of fast-evolving features such as submesoscale eddies and filaments, which play a crucial role in ocean–atmosphere interaction and transport processes.

While recent years have seen major advances in machine learning-based ocean current estimation from satellites, including SST–SSH fusion techniques and physics-informed neural networks^25–28^, GOFLOW represents a fundamental departure. By leveraging the unique temporal resolution of geostationary platforms, orders of magnitude higher than any orbital mission, we circumvent the central limitation that has constrained all previous approaches: sparse temporal sampling.

GOFLOW’s main limitation is the spatial discontinuity created by cloud cover. While the network can process cloudy imagery, treating cloud gradients as input features and masking velocities post-inference, clouds prevent spatio-temporally continuous velocity fields. Remarkably, our validation against shipboard ADCP observations during heavily cloudy conditions (Fig. 3g,h) demonstrates that GOFLOW can achieve excellent agreement with in situ measurements even when operating through intermittent cloud gaps. Although clouds cover approximately 67–72% of the ocean at any instant^61,62^, the high temporal resolution of geostationary imagery allows sufficient surface visibility between clouds for accurate velocity reconstruction. Future work will incorporate microwave radiometer and altimeter observations to fill in the cloud gaps and achieve continuous fields.

Beyond the scientific insight, the implications are far reaching. As next-generation weather and climate models, whether physics-based or driven by artificial intelligence, move towards higher resolutions and shorter forecast timescales, the demand for equally high-resolution observational data becomes critical. GOFLOW meets this need, offering a new class of data products that can be directly assimilated into models or used to validate and refine their outputs. It represents a step forward not only in our capacity to observe the ocean but in our ability to understand and predict its role in Earthʼs system.

## Methods

### Training and inference data

#### Ocean model data

To train GOFLOW, we use the MITgcm LLC4320 simulation, a global, high-resolution (1/48°, ~2 km) ocean model that partially resolves submesoscale dynamics^43^. Our primary requirement for the training dataset is not that the model be perfect at mesoscale circulation everywhere (for example, LLC4320 exhibits known biases in the Gulf Stream separation and mixed-layer depth in this region (for example, refs. ^44,49^)) but that it generate a realistic ensemble of mesoscale and submesoscale flows that advect SST in a statistically comparable way to the real ocean. Recent work comparing the distribution of SST snapshots from LLC4320 with those from infrared satellite sensors (for example, the Advanced Very High Resolution Radiometer and the Visible Infrared Imaging Radiometer Suite) using neural-network-based high-dimensional density estimation has shown a close overlap between the two^67^, supporting this assumption. We therefore use LLC4320 to learn the conditional mapping from short SST-gradient sequences to surface velocity.

For training, we extract 5.3° × 5.3° sub-domains within the 20° N–45° N latitude band in the Atlantic Ocean and work with 256 × 256-point input patches. We use one year of hourly data, with approximately ten sub-domains for training and two for validation. We also experimented with smaller input patches (128 × 128 and 64 × 64 grid points); a summary of their performance is provided in Extended Data Table 1. As discussed in the Patch-size and architecture sensitivity section below, we adopt the 256 × 256 configuration for all results shown here.

#### Geostationary satellite data

For inference, we use Level 1b hourly infrared brightness temperature data (Band 14, 11.2 μm) from the GOES-East Advanced Baseline Imager, which provides 2-km nadir resolution. Three consecutive hourly snapshots are used to predict the velocity field at the central time. As we expect atmospheric correction to the brightness temperature to be a much larger scale contribution, we use the readily available brightness temperature field ABI14.

### Network architecture and training

#### Input representation

We use the logarithm of temperature gradient magnitude, $\log | \nabla T|$, as our input field rather than SST directly. This transformation serves two purposes: it amplifies weak thermal fronts throughout the domain, effectively converting sparse features into a dense field of kinematic tracers and it produces a more Gaussian-like distribution that improves training efficiency, particularly in low-data regimes. Whereas SST could theoretically be used, $\log | \nabla T|$ provides a more direct signature of the velocity field’s kinematic properties, as frontal intensification is explicitly linked to strain and vorticity in the flow.

#### Filtering of target velocities

A key consideration in our training strategy was the treatment of high-frequency, non-advective motions such as internal tides. Because the horizontal advection of SST by such waves is weak, their velocity signature is not strongly encoded in our input tracer fields. Including the full velocity field in the training target would therefore introduce label noise, forcing the network to predict a signal not present in the input. To address this, we pre-processed the target LLC4320 velocity fields by applying an 18-hour low-pass Butterworth filter. This filter effectively removes the semi-diurnal tides (both barotropic and baroclinic components) while retaining the dynamic near-inertial motions and Ekman currents, which can contribute to the advection of SST fronts. Initial experiments trained on unfiltered velocities confirmed this hypothesis, yielding worse performance and demonstrating that the network is probably learning the physically consistent dynamics of temperature advection, rather than merely fitting to a noisy target (not shown). This principled filtering is a crucial distinction from SSH-based methods, where separating balanced flow from ubiquitous internal wave signals remains a primary challenge^68^.

We emphasize that while other wind-induced motions and surface waves can also modulate oceanic SST gradients (for example, ref. ^69^), these effects are typically important at much smaller space and timescales than those being considered here.

#### U-Net architecture

We employ a standard U-Net architecture^42^, an encoder–decoder network with skip connections that excels at image-to-image translation. Its key advantage is that it is fully convolutional, making the network agnostic to input domain size. This allows a model trained on small (256 × 256 points) domains to be applied directly to entire geostationary satellite images (>1,000 × 1,000 points) during inference without tiling or patching.

#### Loss function design

The network is trained by minimizing a composite loss function designed to ensure physical consistency across scales:

1 $${\mathcal{L}}=(1-\lambda ){{\mathcal{L}}}_{{\rm{vel}}}+\lambda {{\mathcal{L}}}_{{\rm{spec}}}.$$

Here ${{\mathcal{L}}}_{{\rm{vel}}}$ controls pointwise velocity accuracy and ${{\mathcal{L}}}_{{\rm{spec}}}$ constrains the kinetic-energy spectrum; varying *λ* trades off these two objectives.

The first term, ${{\mathcal{L}}}_{{\rm{vel}}}$, is the *L*_1_-norm between predicted and target velocity fields; we found that this choice learned faster than an *L*_2_ norm, although this may depend on the details of the hyperparameter search. While essential, this term alone is insufficient to guarantee accuracy at the smallest scales.

The second term, ${{\mathcal{L}}}_{{\rm{spec}}}$, is a log-spectral loss that enforces fidelity at submesoscales by minimizing the mean-squared error between the logarithm of the two-dimensional spatial kinetic energy spectra (computed after windowing the velocity fields using a Tukey window with a 0.2 shape parameter) of the target and predicted velocity fields. Working with the logarithm prevents the largest scales from dominating the loss, so that mesoscale and submesoscale contributions are explicitly constrained. We scanned *λ* over 10 values between 0.05 and 0.9 and selected *λ* = 0.2 as a compromise that substantially improves the small-scale kinetic-energy spectrum (and correspondingly the spatial structure of velocity gradients) while leaving the normalized *L*_1_ velocity error essentially unchanged (changes of order 1%). This rules out the trivial strategy of merely adding red noise, which would broaden the derivatives and increase the pointwise error.

**Extended Data Fig. 8:**
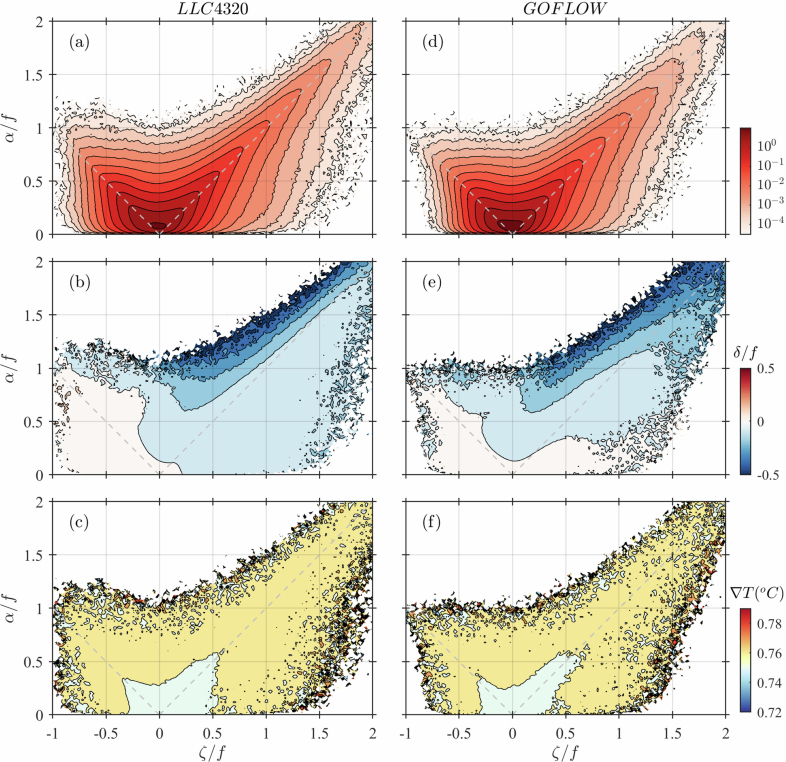
Flow kinematics and statistics computed over the test domain region from GOFLOW reconstruction and LLC4320. (**a**) & (**d**) The joint probability density function (JPDF) of normalized strain (*α*/*f*) vs. normalized vorticity (*ζ*/*f*). The conditional average of (**b**) & (**e**) horizontal divergence (*δ*/*f*) and (**c**) & (**f**) horizontal temperature gradient magnitude (∣ ∇ T∣), for LLC4320 and GOFLOW reconstruction, respectively. We find a remarkably close agreement between the model and GOFLOW estimates even though the U-Net architecture is not trained to minimize PDF losses.

Notably, directly penalizing velocity gradients (vorticity, strain, divergence) alongside velocity did not improve gradient reconstruction compared to velocity loss alone (not shown). The spectral loss proved critical for capturing submesoscale dynamics, as demonstrated by the close agreement in azimuthally averaged spectra between GOFLOW and the reference data (Extended Data Fig. 4) relative to the model trained on joint velocity and velocity-gradient losses (labelled with a ‘gradloss’ tag in Extended Data Figs. 6 and 8). Consistent with this, the configuration selected on the basis of its LLC4320 test performance also provides the best agreement with independent observations when applied to GOES SST gradients, yielding the lowest errors against NESMA shipboard ADCP and surface drifter velocities (Extended Data Fig. 1).

#### Training protocol

Starting training directly with the full joint loss in equation (1) and *λ* > 0 often resulted in poor or no learning. We therefore adopt a simple curriculum: we first train using only the velocity loss (that is, *λ* = 0) for 100 epochs, during which the network already reproduces the large-scale part of the kinetic-energy spectrum and then fine-tune for an additional 100 epochs with the joint loss (*λ* = 0.2). In this second stage, the spectral term acts as a regularizer on ${{\mathcal{L}}}_{{\rm{vel}}}$, selectively sharpening mesoscale and submesoscale structure while keeping the pointwise velocity error close to that of the pure-${{\mathcal{L}}}_{{\rm{vel}}}$ model.

We employ cosine annealing with warm restarts^70^, cyclically decreasing the learning rate from 10^−3^ to zero over five epochs before resetting. Model selection uses the global minimum of ${{\mathcal{L}}}_{{\rm{vel}}}$ on the test set across the entire training run, ensuring that we capture the most performant model state in terms of pointwise velocity accuracy^71^.

#### Patch-size and architecture sensitivity

We also examined how the choice of input patch size affects model performance. Keeping the architecture and training protocol fixed, we trained U-Net variants on 256 × 256, 128 × 128 and 64 × 64 input patches and evaluated skill on the held-out test set Extended Data Table 1. With the pure velocity loss (*λ* = 0), all three U-Net configurations achieve similar *R*^2^ values for the zonal and meridional velocities, indicating that the bulk of the large-scale mapping from SST gradients to velocity is not strongly sensitive to patch size in this regime. When the joint velocity-spectral loss (*λ* = 0.2) is used, the 256 × 256 model retains essentially the same velocity accuracy as the pure velocity-loss case, whereas the 128 × 128 and 64 × 64 models show a modest degradation in *R*^2^. This degradation coincides with a reduced ability of the spectral term to match the small-scale kinetic-energy spectra on smaller, non-periodic patches and is therefore most naturally interpreted as a finite-domain/spectral estimation effect rather than a clear signature of spatial locality. For this reason, we use 256 × 256 patches and *λ* = 0.2 for the main GOFLOW results.

#### Exploratory physics-informed inversion attempts

In addition to the U-Net approach, we explored more explicitly physics-informed inversion strategies to clarify what the network might be learning and to assess alternative ways of recovering velocity from SST.

As a first step, we formulated a discrete forced advection–diffusion equation as a linear inverse problem for the velocity field, given consecutive SST snapshots and prescribed forcing. In practice, the resulting system is large, dense and highly ill-conditioned, and even with standard regularization the obtained velocity fields had high degree of noise that had little coherence or skill with the ground-truth LLC4320 solution.

We then tested an implicit neural-network formulation^72^, conceptually similar to physics-informed neural networks^73^. Each velocity component was represented by a fully connected network of space and time; such a continuous field representation of data is referred to as a Neural Field^74^; surface forcing is taken from the atmospheric product used to drive LLC4320 (ERA-Interim) and finite differences are used to approximate spatial and temporal gradients in the temperature equation. The residual of the forced advection–diffusion equation was added as a loss term. On the LLC4320 data, this approach converged and captured the large scales, but it was substantially slower and overall less accurate than the U-Net, particularly at the smaller scales of interest. Applying this implicit-physics framework directly to GOES imagery proved even more difficult. Speckle-like noise in the satellite product, especially in the time-derivative (temperature tendency), led to large, noisy equation residuals, and denoising attempts did not resolve the issue. In contrast, the U-Net appears relatively robust to this type of noise, implicitly downweighting incoherent small-scale features in SST while retaining the coherent frontal signals.

### Validation on held-out simulations

To assess GOFLOW’s ability to reconstruct ocean dynamics, we performed comprehensive validation on held-out LLC4320 domains. The results demonstrate remarkable fidelity across multiple metrics:

Direct field comparisons show promising snapshot agreement for vorticity (*ζ*/*f*), strain (*α*/*f*) and horizontal divergence (*δ*/*f*), a quantity strongly enhanced at submesoscales (Extended Data Fig. 2). Although *δ*/*f* is somewhat less accurately reproduced than the strain and the vorticity, it still shows the expected spatial correlations, namely: strong convergence in cyclonic regions. Most importantly, GOFLOW’s ability to estimate the divergence field is an important advancement, as it cannot be inferred from traditional geostrophic approaches.

Spectral characteristics are preserved with high accuracy. The kinetic energy, vorticity, divergence and strain variance spectra closely match the ground truth across nearly two decades of wavenumbers (Extended Data Fig. 4a–d) with only a slight reduction in variance (a larger reduction is seen in winter compared to summer; Extended Data Fig. 5). Remarkably, despite training only on spatial spectra, GOFLOW accurately reproduces the frequency spectra (Extended Data Fig. 4e–h) and full frequency–wavenumber characteristics (Extended Data Fig. 3), suggesting that the learned mapping also preserves the temporal variability of the LLC4320 solution, even though no explicit temporal constraints were imposed in the loss function.

Statistical distributions show GOFLOW captures the characteristic hallmarks of submesoscale turbulence, namely the positive vorticity skewness and negative divergence skewness (Extended Data Fig. 6). While there is a slight reduction in variance, the shapes of the probability density functions are remarkably well preserved, including the heavy tails indicative of intense submesoscale events, evinced by the close match of the variance normalized PDFs.

### Emergent properties not explicitly modelled

A striking aspect of GOFLOW is its ability to capture dynamical relationships that were not explicitly included in the training loss, as follows.

#### Flow topology relationships

The joint PDFs of strain versus vorticity, and their conditional relationships with divergence and temperature gradients, are remarkably well reproduced (Extended Data Fig. 8). These correlations, fundamental to submesoscale dynamics, emerge naturally from the spectral constraint without explicit training.

#### Temporal dynamics

Although trained on individual snapshots with only spatial spectral constraints, GOFLOW accurately captures the temporal evolution of submesoscale features, as evidenced by the frequency spectra and frequency-wavenumber spectra (Extended Data Figs. 3 and 4).

These emergent properties suggest that the spectral loss function, by enforcing the correct energy distribution across scales, implicitly constrains the network to learn physically consistent dynamics beyond what is explicitly supervised.

### Independent validation with observations

For regions with cloud cover, GOFLOW processes the logarithm of cloud gradients as input features, with velocity masking applied post-inference to remove cloud-contaminated areas. This approach enabled successful validation even during cloudy conditions.

- AVISO Segment Sol multimissions d’ALTimétrie, d’Orbitographie et de localisation précise (SSALTO)/Data Unification and Altimeter Combination System (DUACS) products were processed by SSALTO/DUACS and distributed by AVISO+ (https://www.aviso.altimetry.fr) with support from Centre National d’Études Spatiales (CNES). We used geostrophic currents (0.25°, daily) as a basin-scale baseline.
- SWOT L3 Expert product (2 km along track, 21-day repeat) provided mesoscale comparison along spring 2023 Gulf Stream tracks.
- Copernicus Marine Environment Monitoring Service Global Ocean in situ observations (product INSITU_GLO_PHY_UV_DISCRETE_MY_013_044): a representative set of drifter data across all seasons (2022/12; 2023/01; 2023/04; 2023/05; 2023/08; 2023/10).
- Shipboard ADCP observations: 300-kHz acoustic Doppler current profiler data from multiple research cruises (EN-702B, 06/2023; EN-66, 01/2021; AT-49, 06/2022, AR74, 06/2023; AR-72A, 04/2023; AR72-B, 05/2023; AR-71, 01/2023 R/V Armstrong AR-73 cruise during NESMA 2023 (15–16 May 2023), all using the 20–25 m depth range).

### Limitations

A fundamental limitation is the dependence on the training prior: the current framework uses a single year of LLC4320 data from a limited Atlantic region. The effective resolution and dynamics that GOFLOW can confidently resolve in the real world are bounded by the accuracy and resolution of this training simulation (~2 km, partially resolving submesoscales). Subgrid-scale parameterizations and model-specific biases in LLC4320 may propagate into the GOFLOW product, though the extent of this effect remains to be quantified. This limitation motivates broader, multi-year, high-resolution regional modelling efforts specifically designed to support machine learning-based inversion methods.

The current framework assumes Euclidean geometry in its convolutional operations, valid for the mid-latitude 5.3° × 5.3° sub-domains used here but preventing direct application of mid-latitude trained models to global, pole-to-pole data. Future work will incorporate positional encodings to make the network aware of Earth’s curvature. In fact, training over a much wider range of latitudes actually decreases model accuracy against the NESMA shipboard observations, supporting the need for positional information in the U-Net inputs.

A central achievement of this study is the observation of more accurate velocity gradients than traditional products (for example, Fig. 3a,b). However, as Extended Data Figs. 2 and 6 show, GOFLOW under predicts high-velocity-gradient magnitudes in comparison to LLC4320. Whereas the GOFLOW product shows good agreement with measured velocities during the NESMA campaign and comparison with drifter and SWOT products, an extensive evaluation across different regions and multiple years is underway and will be the focus of subsequent studies. The comparison with LLC4320 shows more accurate results during summer months relative to winter months, something that is currently being addressed with more data generation at similar resolution in the region through multi-year submesoscale-permitting regional simulations. These additional simulations would also help quantify the dependence of the learned velocity fields on model-specific biases.

Whereas GOFLOW can process cloudy imagery, as demonstrated by our successful NESMA validation during heavily cloudy conditions where we applied the network to cloud gradient features and subsequently masked the velocity output, the primary limitation is that clouds create spatial discontinuities in the velocity field. Future work will incorporate microwave radiometer and altimeter observations to provide spatio-temporally continuous fields even under persistent cloud cover. This will also improve the quality of the GOES cloud detection and masking algorithms that can lead to incorrect predictions if clouds are not captured accurately.

Another missing ingredient in the current work is the lack of systematic uncertainty estimation that consists of aleatoric (that is, pertaining to the complexity of the data for prediction, for example, winter vs summer) and epistemic (that is, uncertainty in the modelling) uncertainties^75^. While the latter can be obtained through multiple ensembles of U-Nets trained on the same data^76^, the former is more challenging and requires specialized training^77^. Future work will address this issue.

## Online content

Any methods, additional references, Nature Portfolio reporting summaries, source data, extended data, supplementary information, acknowledgements, peer review information; details of author contributions and competing interests; and statements of data and code availability are available at 10.1038/s41561-026-01943-0.

## Supplementary information

Supplementary Video 1 Temporal evolution of the fields presented in Fig. 2 to illustrate the unique ability of GOFLOW to capture the spatio-temporal variability of submesoscale features in the Gulf Stream region.

## Figures and Tables

**Extended Data Table 1:**
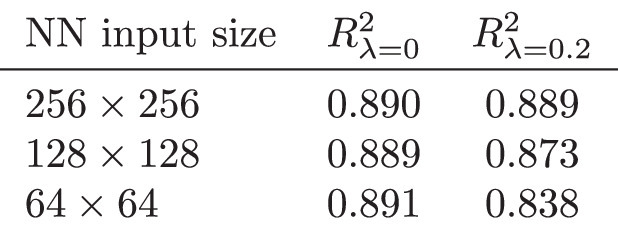
Coefficient of determination (r^2^) for U-Net variants trained with different input patch sizes on the LLC4320 test set, comparing pure velocity-loss training (*λ* = 0) and joint velocity–spectral loss (*λ* = 0.2)

.

## Data Availability

All presented data and GOFLOW products are available via Zenodo at 10.5281/zenodo.15815704 (ref. ^78^). The SWOT Level 3 data are available at 10.24400/527896/a01-2023.018. AVISO products were processed by SSALTO/DUACS and distributed by AVISO+ (https://www.aviso.altimetry.fr) with support from CNES. The R/V Armstrong current observations are available through the Rolling Deck to Repository (R2R) for the NESMA 2023 cruise number AR75 at 10.7284/156607. The MITGCM LLC4320 ocean model surface data were obtained from the Pangeo forge catalogue at https://catalog.pangeo.io/browse/master/ocean/LLC4320/. Land topography shown in the figures is from the ETOPO 2022 15 Arc-Second Global Relief Model National Oceanic and Atmospheric Administration (NOAA) National Centers for Environmental Information; 10.25921/fd45-gt74). GOES satellite imagery was obtained via the NOAA GOES Open Data Registry on Amazon Web Services (AWS) at https://registry.opendata.aws/noaa-goes.

## Code availability

All training, testing and inference scripts are written in Python and are available via GitHub at https://github.com/ksr-ocean/goflow. Colourmaps were generated using the MATLAB function brewermap, which implements the ColorBrewer palettes developed by Cynthia Brewer, Mark Harrower and The Pennsylvania State University.
